# Supplementary material for: Structure and assembly of scalable porous protein cages
Source: Nat Commun. 2017 Mar 10;8:14663. doi: 10.1038/ncomms14663 (PMC5354205; doi:10.1038/ncomms14663)
Supplement: Supplementary Information — Supplementary Figures and Supplementary Tables. [file ncomms14663-s1.pdf]

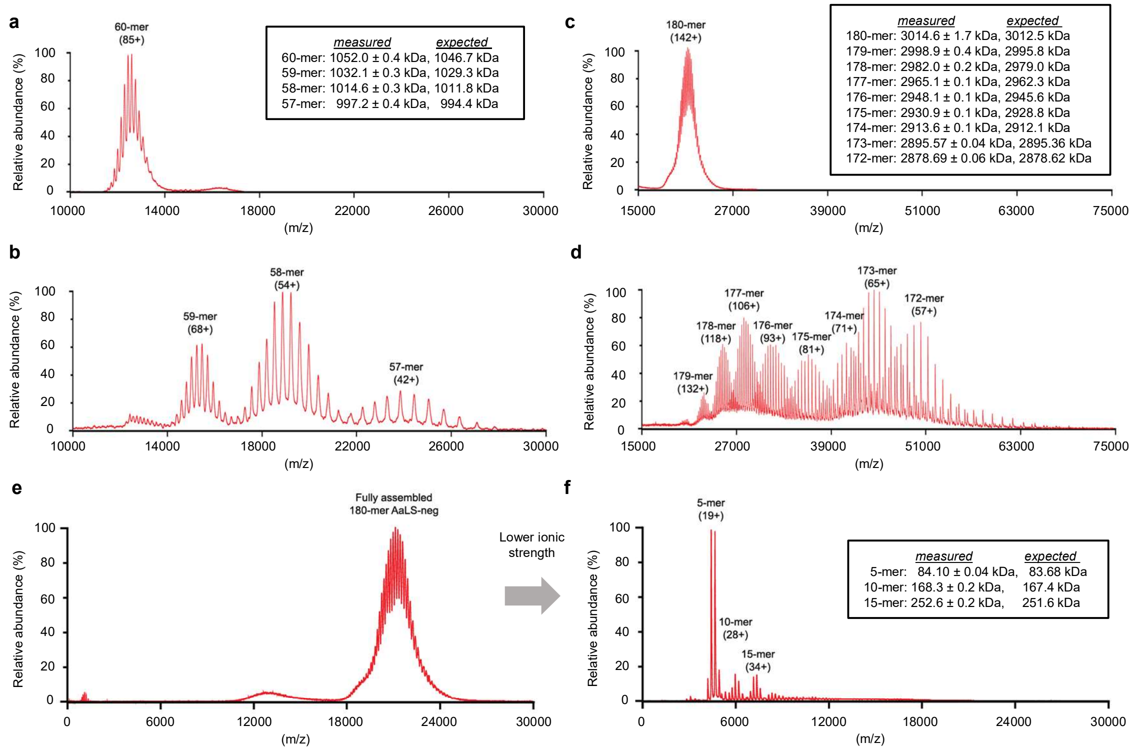

Supplementary Figure 1 │ Tandem MS analysis of AaLS-wt and AaLS-neg. a,b, Intact particles of AaLS-wt were mass-selected (**a**) and fragmented by collision-induced dissociation (CID) (**b**). The most abundant charge states are annotated. The measured and expected masses are shown in the insets. CID causes sequential loss of monomeric AaLS subunits carrying a high amount of charge. The concomitant particles that have lost a varying number of the AaLS monomeric subunits appear at increasingly higher mass to charge ratios. For AaLS-wt, particles with 59, 58, and 57 copies of the monomer were detected, confirming the oligomeric state of the intact precursor as being 60-mer. **c,d,** Intact particles of AaLS-neg were mass-selected (**c**) and fragmented by collision-induced dissociation (CID) (**d**). For AaLS-neg, particles with 179 down to 172 copies of the monomer were detected, confirming the oligomeric state of the intact particle to be 180-mer. **e,f,** Disassembly of the AaLS-neg cage. Fully assembled 180-mer AaLS-neg cages (**e**) were disassembled into pentameric capsomers and higher-order oligomers of 10 and 15 copies of the AaLS-neg monomer in 10 mM ammonium acetate, pH 7.8 (**f**). The most abundant charge states are annotated. The measured and expected masses are shown in the inlet. Notably, no hexamers, trimers or dimers were detected.

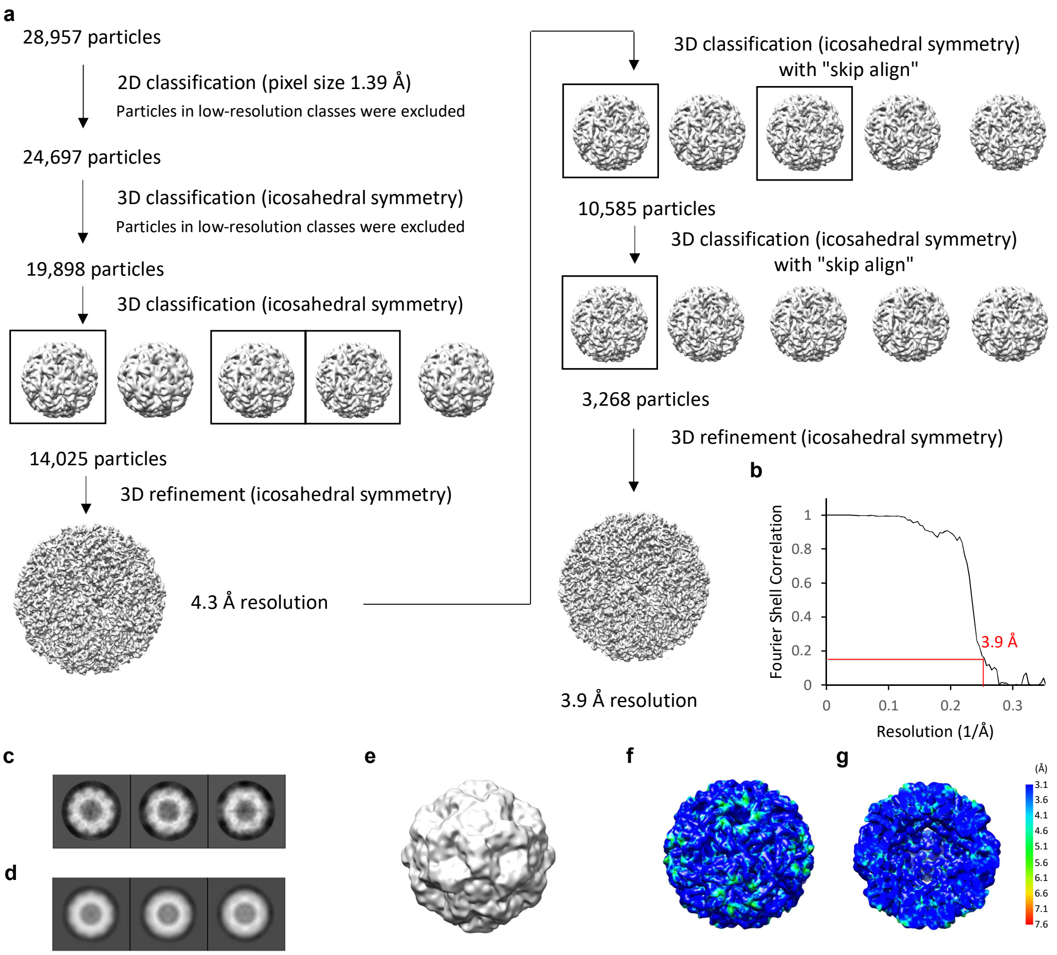

Supplementary Figure 2 │ Cryo-EM single-particle reconstruction of AaLS-wt. **a,** Workflow of computational sorting. Particles in boxed classes were used for further refinement. **b,** The "gold-standard" Fourier shell correlation (FSC) curve, which estimates the resolution of the final EM density map at 3.9 Å according to the FSC = 0.143 criterion. **c,d,** Selected 2D class-averages (**c**) and 2D projections of an initial model corresponding to each class-average (**d**). **e,** A 3D-view of the initial model. **f,g,** Local resolution maps of a whole cage (**f**) and a half-sliced cage (**g**) that visualize the outer and inner surface of the cage, respectively.

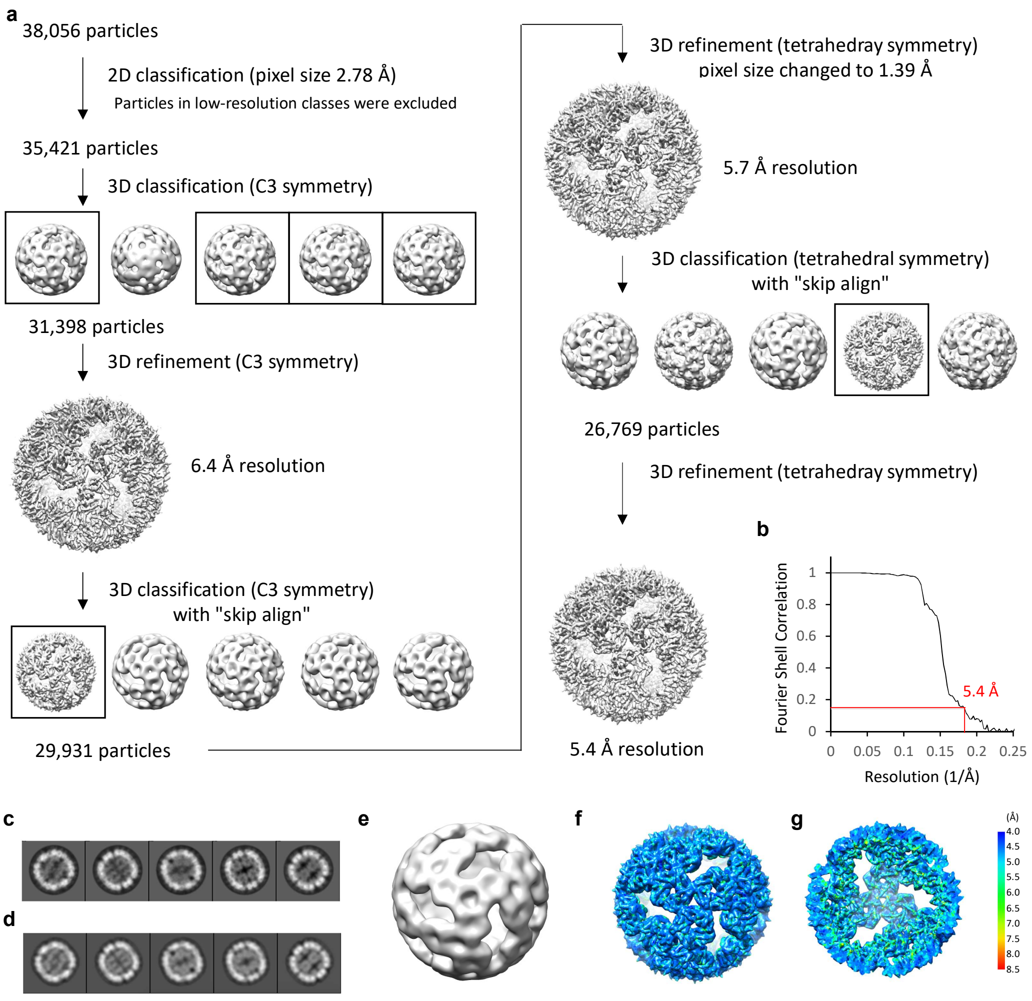

Supplementary Figure 3 │ Cryo-EM single-particle reconstruction of AaLS-neg. a, Workflow of computational sorting. Particles in boxed classes were used for further refinement. **b,** The “gold-standard” Fourier shell correlation (FSC) curve, which estimates the resolution of the final EM density map at 5.4 Å according to the FSC = 0.143 criterion. **c,d,** Selected 2D class-averages (**c**) and 2D projections of an initial model corresponding to each class-average (**d**). **e,** A 3D-view of the initial model. **f,g,** Local resolution maps of a whole cage (**f**) and a half-sliced cage (**g**) that visualize the outer and inner surface of the cage, respectively.

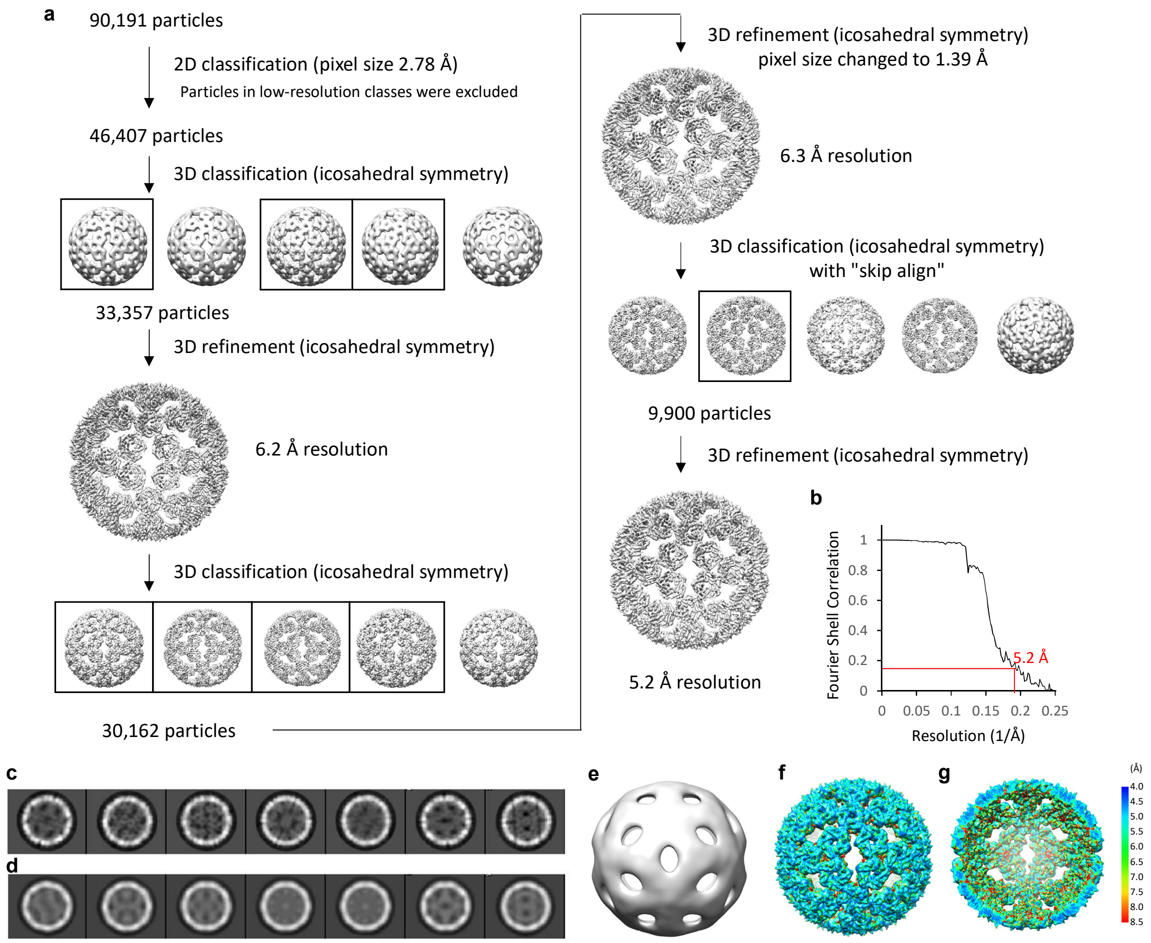

Supplementary Figure 4 │ Cryo-EM single-particle reconstruction of AaLS-13. **a,** Workflow of computational sorting. Particles in boxed classes were used for further refinement. **b,** The "gold-standard" Fourier shell correlation (FSC) curve, which estimates the resolution of the final EM density map at 5.2 Å according to the FSC = 0.143 criterion. **c,d,** Selected 2D class-averages (**c**) and 2D projections of an initial model corresponding to each class-average (**d**). **e,** A 3D-view of the initial model. **f,g,** Local resolution maps of a whole cage (**f**) and a half-sliced cage (**g**) that visualize the outer and inner surface of the cage, respectively.

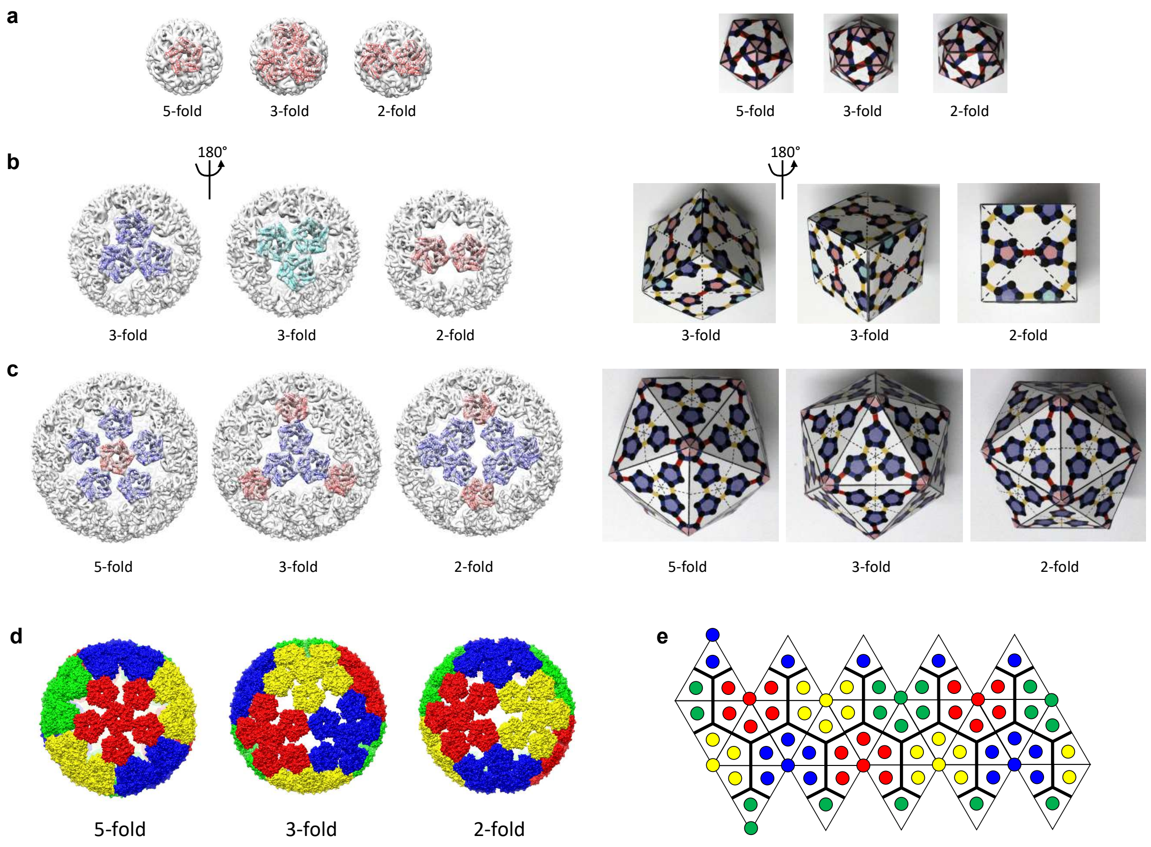

Supplementary Figure 5 │ Symmetry of the AaLS variants. a–c, Low-pass filtered cryo-EM density maps of AaLS variants with fitted pentamer models and the corresponding paper models created from 2D representation of the cages shown in Fig. 2. Icosahedral 5-, 3-, and 2-fold symmetrical views are shown for AaLS-wt (**a**) and AaLS-13 (**c**) while tetrahedral 3- and 2-fold symmetrical views are shown for AaLS-neg (**b**). Symmetrically non-equivalent pentamers are shown in different colors in each AaLS variant. **d,** AaLS-13 cages viewed as an extended dodecahedron (12 × 30-mer). Twelve faces of a dodecahedron, each of which is constituted of a pentamer associated with five other pentamers, are explicitly shown in different colors. Note that dodecahedral symmetry is equivalent to icosahedral symmetry. **e,** 2D representation of the AaLS-13 cage with an emphasis of building blocks for the extended dodecahedron. Each filled circle represents a pentamer, and 6 neighboring pentamers shown in the same color form one of the twelve dodecahedral faces.

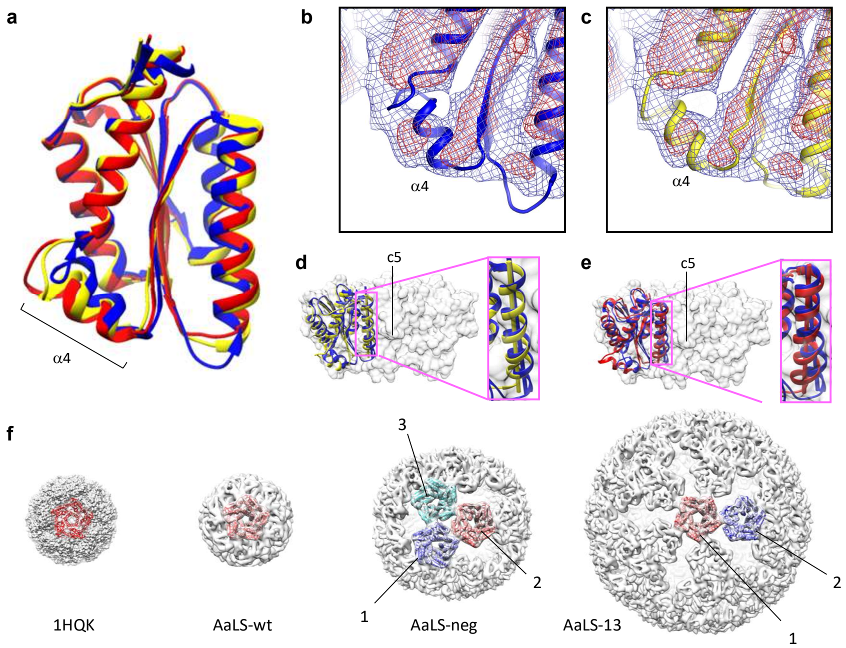

Cα RMSD (Å)

	1HQK (red)	Wt (red)	neg-1 (blue)	neg-2 (red)	neg-3 (cyan)	13-1 (red)	13-2 (blue)
1HQK (red)		0.52 Å	2.1 Å	2.3 Å	2.1 Å	2.8 Å	2.8 Å
Wt (red)			2.0 Å	2.1 Å	2.0 Å	2.8 Å	2.7 Å
neg-1 (blue)				0.74 Å	0.83 Å	1.5 Å	1.2 Å
neg-2 (red)					0.70 Å	1.3 Å	1.2 Å
neg-3 (cyan)						1.5 Å	1.4 Å
13-1 (red)							1.1 Å
13-2 (blue)							

Supplementary Figure 6 │ Superposition of the AaLS monomers and pentamers. a, Superposition of the AaLS-wt monomer (blue, 1HQK) and representative monomers of the refined AaLS-neg (yellow) and AaLS-13 (red) models. While the overall fold is conserved, the α4 helix is flexibly repositioned to adapt to different inter-pentamer arrangements. **b,c,** Close-up view of the cryo-EM density map of the α4 helix in an AaLS-neg monomer fitted with the original AaLS-wt model (**b**) and after refinement (**c**). The EM density is contoured at 2.0 (slate) and 3.5 (red) sigma levels, respectively. **d,e,** The refined models of the AaLS-neg pentamer (**d**) or the AaLS-13 pentamer (**e**) were superimposed onto the crystal structure pentamer of AaLS-wt (1HQK). For clarity, only one monomer for each structure is shown as ribbon diagram (AaLS-wt, blue; AaLS-neg, yellow; and AaLS-13, red). The AaLS-wt pentamer is shown as a transparent surface. The mutant pentamers are slighty tilted outwards from the 5-fold rotational symmetry axes. **f,** Cα RMSDs of the AaLS pentamer models. Symmetrically non-equivalent pentamers in the AaLS-neg and AaLS-13 structures are presented in different colors in each structure. The RMSD was calculated for 770 Cα atoms from each pentamer (residues 1–154). The resulting RMSD values are shown in the table.

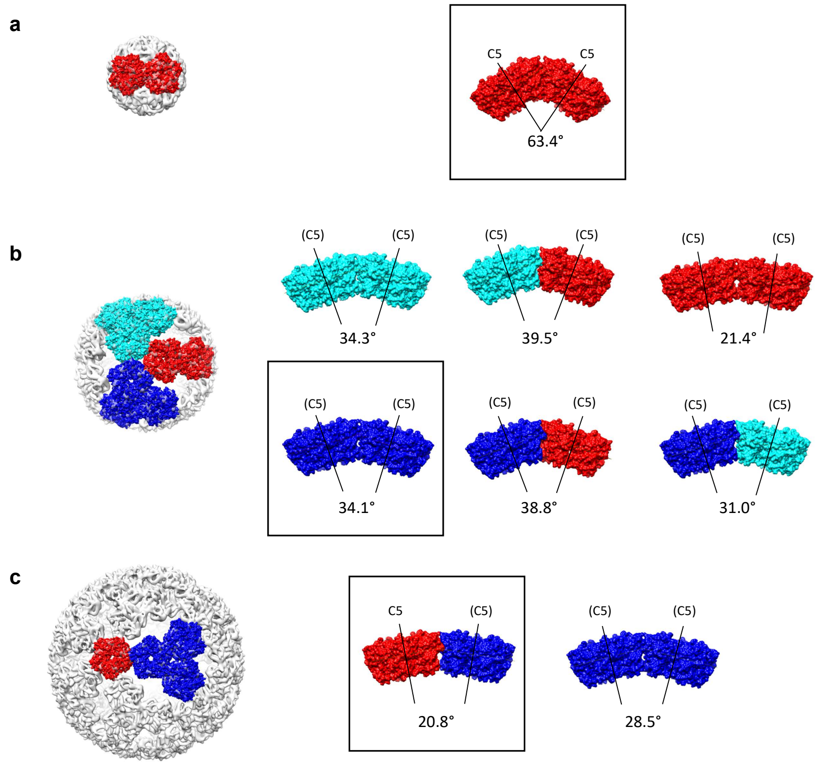

Supplementary Figure 7 │ Angles between local (pseudo) 5-fold rotational symmetry axes of AaLS-wt (a), AaLS-neg (b), and AaLS-13 (c) Local (pseudo) 5-fold rotational symmetry axes of adjacent petamers and angles between the corresponding axes are shown. For the AaLS-neg and AaLS-13 structures, symmetrically non-equivalent pentamers are presented in the same color scheme as shown in supplementary Fig. 5a–c. The represented structures shown in Fig. 6c are boxed.

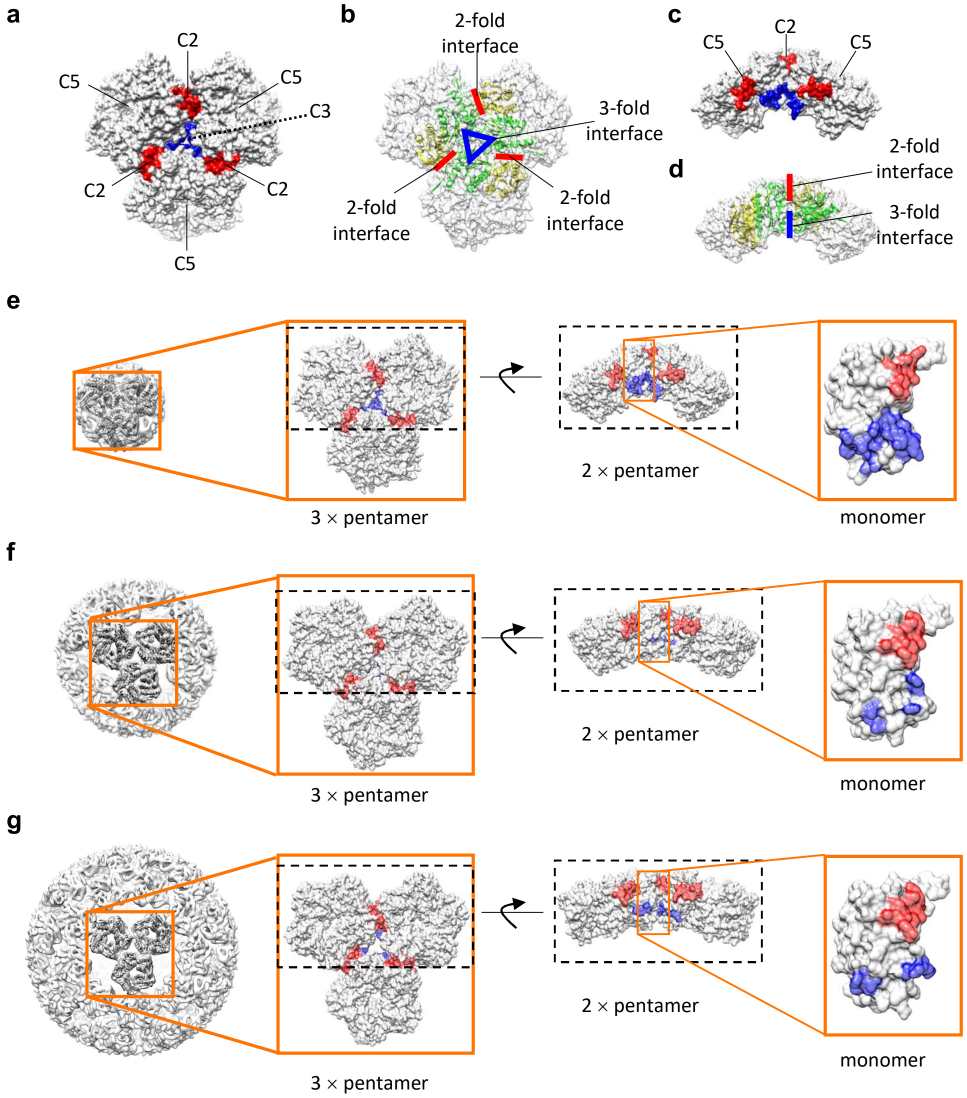

Supplementary Figure 8 │ Interpentamer interfaces of the AaLS variants. a, Three adjacent AaLS-wt pentamers (1HQK) around a 3-fold rotational symmetry axis. Estimated contact areas (C_α–C_α distance <8 Å) around 3- and 2-fold rotational symmetry axes of the icosahedral lattice are shown in blue and red, respectively. **b,** The same view schematically showing the 3-fold and 2-fold interfaces by blue and red bars, respectively. Monomers that form the presented interfaces are shown as ribbon diagrams in green and yellow. **c,d,** Two adjacent AaLS-wt pentamers (1HQK) around a 2-fold rotational symmetry axis. Estimated contact areas (C_α–C_α distance <8 Å) around 3- and 2-fold rotational symmetry axes of the icosahedral lattice are similarly colored (**c**) and schematically shown (**d**) as **a** and **b**, respectively. **e–g,** Interpentamer interfaces around 3-fold rotational symmetry axes of AaLS-wt (**e**), AaLS-neg (**f**), and AaLS-13 (**g**). Estimated contact areas (C_α–C_α distance <8 Å) originate in the 3-fold and 2-fold interfaces of the wt structure are shown in blue and red, respectively.

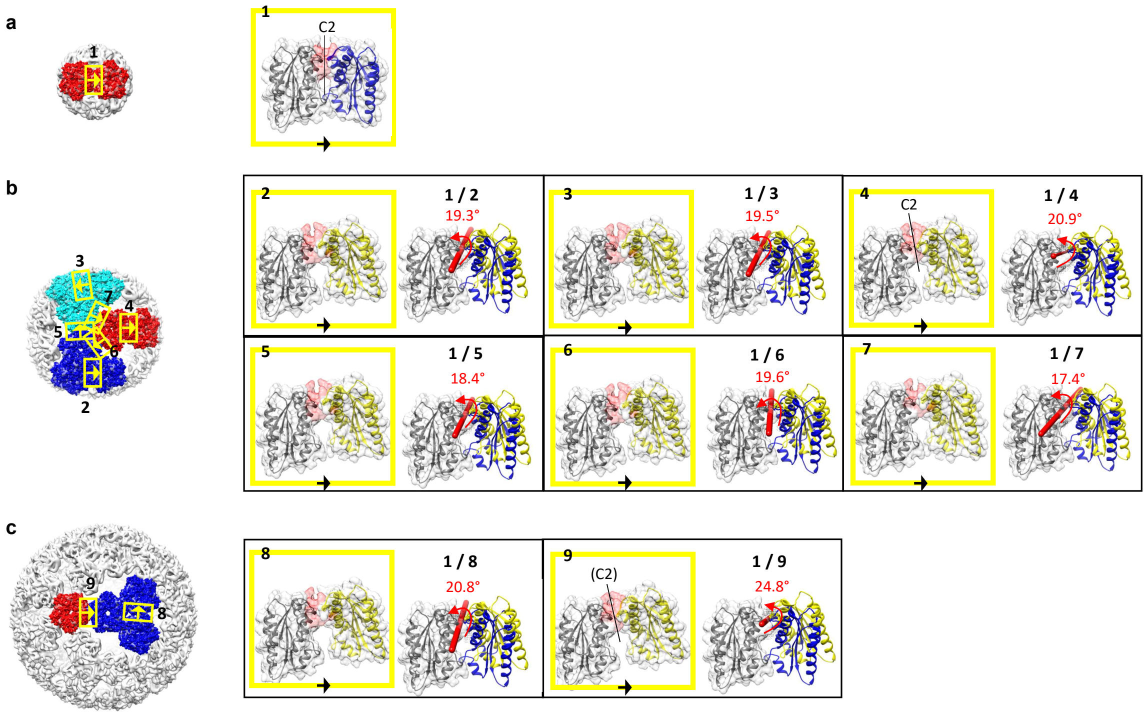

Supplementary Figure 9 │ Hinge motion around the 2-fold interfaces. **a,** Two adjacent monomers that are involved in inter-pentamer interfaces around the 2-fold rotational symmetry axis are shown for AaLS-wt. The corresponding monomers are boxed in yellow with an arrow and an interface number (**1**) from the cage structure. Ribbon diagrams of the yellow-boxed monomers, which were created by rigid-body fitting of the monomers in the 1HQK crystal structure to the EM density maps using the "Fit in Map" function in UCSF Chimera[4], are shown respectively in grey and blue, corresponding to the arrow tail and arrowhead in the cage structure to the left. The 2-fold contact area (C_α–C_α distance <8 Å) is shown in red. **b,** Six symmetrically non-equivalent inter-pentamer interfaces in the AaLS-neg structure. Each is boxed in yellow with an arrow and the relevant interface (**2**–**7**) from the capsid structure. Ribbon diagrams of the yellow-boxed monomers were created as described for **a**. The grey monomer of each structure was superimposed onto that of wt (**1**). The rotational axis and angle of the other monomer (yellow) with respect to the wt monomer (blue) were calculated using the "measure rotation" function in UCSF Chimera and are shown in red. **c,** Two symmetrically non-equivalent inter-pentamer interfaces in the AaLS-13 structure. Both are boxed in yellow with an arrow and interface numbers (**8** and **9**) from the assembled cage shown to the left. Ribbon diagrams of the yellow-boxed monomers were created as described for **a** and superimposed onto **1** as described in **b**.

Supplementary Table 1 │ Homomer structures with tetrahedral, octahedral, or icosahedral symmetry reported in the PDB.

Tetrahedral (homomer: 272 structures)

stoichiometry	number of structures
Homo 12-mer	265
Homo 24-mer	6
Homo 180-mer	1[a]

Octahedral (homomer: 290 structures)

stoichiometry	number of structures
Homo 24-mer	288
Homo 48-mer	2

Icosahedral (homomer: 279 structures

stoichiometry	number of structures
Homo 60-mer	109[b]
Homo 120-mer	10
Homo 180-mer[c]	105
Homo 240-mer	10
Homo 300-mer	1
Homo 360-mer	10[d]
Homo 420-mer	24
Homo 480-mer	2
Homo 540-mer	1
Homo 720-mer	4
Homo 780-mer	3

[a] This work (AaLS-neg). The AaLS-neg cage has the highest copy number of a single subunit (180-mer) among all the reported structures possessing tetrahedral symmetry.
[b] Including this work (AaLS-wt).
[c] Protein stoichiometry analysis with >119,600 structures reported in PDB showed that all homo180-mer structures possess icosahedral symmetry except AaLS-neg determined in this work.
[d] Including this work (AaLS-13). See also Supplementary Table 2.

Supplementary Table 2 │ Homo 360-mer structures reported in PDB.

entry ID	name	released year	method	resolution (Å)	symmetry
1SVA	simian virus 40	1996	X-ray diffraction	3.10	icosahedral (T = 7d)[a]
1SIE	murine polyomavirus complexed with a disialylated oligosaccharide	1996	X-ray diffraction	3.65	icosahedral (T = 7d)[a]
1SID	murine polyomavirus complexed with 3'sialyl lactose	1996	X-ray diffraction	3.65	icosahedral (T = 7d)[a]
3IYJ	bovine papillomavirus type 1 outer capsid	2010	EM	4.20	icosahedral (T = 7d)[a]
3IYH	P22 procapsid coat protein	2010	EM	8.20	icosahedral (T = 7l)[b]
3IYS	avian polyomavirus	2011	EM	11.30	icosahedral (T = 7d)[a]
3J6R	human papillomavirus Type 16 capsid	2014	EM	9.10	icosahedral (T = 7d)[a]
5FUA	BK polyomavirus	2016	EM	7.60	icosahedral (T = 7d)[a]
5JB1	human papillomavirus type 59 L1 virus-like particle	2016	EM	6.00	icosahedral (T = 7d)[a]
ZZZZ	AaLS-13	2016[c]	EM	5.4	icosahedral[d]

[a] Pentameric capsomers are located in both 5- and 6-coordinated positions of a T = 7d icosahedral lattice (5-mer × 72 = 360-mer).
[b] Pentameric capsomers at 5-coordinated position of a T = 7l icosahedral lattice are lacked (6-mer × 60 = 360-mer).
[c] This work.
[d] Pentameric capsomers are arranged in an icosahedral lattice, but the global structure is not skewed (5-mer × 72 = 360-mer). See also Fig. 4 and Supplementary Fig. 5d,e.
